# Content of selected elements and low-molecular-weight organic acids in fruiting bodies of edible mushroom *Boletus badius* (Fr.) Fr. from unpolluted and polluted areas

**DOI:** 10.1007/s11356-016-7222-z

**Published:** 2016-07-28

**Authors:** Mirosław Mleczek, Zuzanna Magdziak, Monika Gąsecka, Przemysław Niedzielski, Pavel Kalač, Marek Siwulski, Piotr Rzymski, Sylwia Zalicka, Krzysztof Sobieralski

**Affiliations:** 1 Department of Chemistry, Poznan University of Life Sciences, Poznań, Poland; 2 Faculty of Chemistry, Adam Mickiewicz University in Poznań, Poznań, Poland; 3 Department of Applied Chemistry, Faculty of Agriculture, University of South Bohemia, České Budějovice, Czech Republic; 4 Department of Vegetable Crops, Poznan University of Life Sciences, Poznań, Poland; 5 Department of Biology and Environmental Protection, University of Medical Sciences, Poznan, Poland

**Keywords:** Edible mushroom, *Boletus badius*, Underlying substrate, Contamination, Mineral composition, Flotation tailings, Soil, Organic acids

## Abstract

The aim of the study was to (i) investigate the potential of edible mushroom *Boletus badius* (Fr.) Fr. to accumulate 53 elements from unpolluted acidic sandy soil and polluted alkaline flotation tailing sites in Poland, (ii) to estimate the low-molecular-weight organic acid (LMWOA) profile and contents in fruit bodies, and finally (iii) to explore the possible relationship between elements and LMWOA content in mushrooms. The content of most elements in fruiting bodies collected from the flotation tailings was significantly higher than in mushrooms from the unpolluted soils. The occurrence of elements determined in fruiting bodies of *B. badius* has been varied (from 0.01 mg kg^−1^ for Eu, Lu, and Te up to 18,932 mg kg^−1^ for K). The results established the high importance of element contents in substrate. Among ten organic acids, nine have been found in wide range: from below 0.01 mg kg^−1^ for fumaric acid to 14.8 mg g^−1^ for lactic acid. Lactic and succinic acids were dominant in both areas, and citric acid was also in high content in polluted area. The correlation between element contents and the individual and total content of LMWOAs was confirmed.

**Electronic supplementary material:**

The online version of this article (doi:10.1007/s11356-016-7222-z) contains supplementary material, which is available to authorized users.

## Introduction

*Boletus badius* (Fr.) Fr. (current name: *Imleria badia* (Fr.) Vizzini) is one of the most common, widely consumed edible wild mushroom species in European forests. Its ability to accumulate various elements in fruiting bodies collected from both unpolluted (Borovička and Řanda 2007; Malinowska et al. 2004; Rudawska and Leski 2005; Svoboda and Chrastný 2008) and polluted areas (Svoboda et al. 2006; Niedzielski et al. 2013) has been described in numerous studies (Kojta et al. 2012; Malinowska et al. 2004; Mleczek et al. 2015a; Reczyński et al. 2013). It is worth underlining that this species is capable of growing on extremely polluted sites (Mleczek et al. 2015b); however, trace element contents were usually reported in fruiting bodies collected from various unpolluted areas. Unfortunately, very few works comparing *B. badius* fruiting bodies collected from significantly different ecosystems as regards their ability to accumulate various elements are available (Kojta et al. 2012; Mleczek et al. 2015b). Data on multielemental analysis of *B. badius* are very scarce (Falandysz et al. 2001). According to a review of Kalač (2010), there are limited data on the content of selected elements such as B, Ga, Li, Tl, or Sn. One reason for this scarcity is a lack of analytical possibilities (Falandysz 2008). Additionally, the question as to the significance of pH/Eh conditions in element accumulation in mushroom fruiting bodies has yet to be explained, because such information is minimal (Falandysz and Borovička 2013). An increase/decrease of substrate pH modulates the bioavailability of most elements; therefore, the low-molecular-weight organic acids present in plants (Kutrowska and Szelag 2014) can play a significant role, where their focus is shifted to the influence of elements on defense mechanism (detoxicative and antioxidative), metal chelation, accumulation, and sequestration. However, to our knowledge, such information in relation to mushroom bodies has yet to be elucidated, and currently, information regarding the acids present in mushrooms only relates to their antioxidant properties or their nutritional value data.

Mushrooms contain organic acids (Ribeiro et al. 2006; Barros et al. 2013); however, their relation to trace element accumulation in fruiting bodies has not yet been studied. In plants, the content of low-molecular-weight organic acids (LMWOAs) varies considerably among species and cultivars (biotic factors), and it is also influenced by local (abiotic) factors such as soil, climate, pollution, etc. (Nawirska-Olszańska et al. 2014). Extremely adverse growth conditions, such as high concentration of metals in soil, low nutrient contents, low/high humidity, acidification, or salinity, initiate a defense mechanism, which promotes survival under difficult environmental conditions. Efficient ways to reduce the toxicity of metals present in soil include both a range of extracellular strategies, which involve reducing metal uptake by immobilizing them in the soil matrix, and transport of metals to the aerial part of plants. However, when metals get into the cell, intracellular mechanisms are activated based on metal-ligand complexation, where phytochelatins, LMWOAs, are used for the compartmentation of complexes into the vacuole. LMWOAs, such as oxalic, citric, malonic, succinic, and/or tartaric acids exudated by plants (Dresler et al. 2014) or mushrooms (Ousmanova and Parker 2007), have been known to have a capacity to remove heavy metals from polluted soil. However, to date, studies evaluating possible relationships between LMWOAs in mushrooms and trace element concentrations are scarce.

The aim of the study was to compare the ability of *B. badius* fruiting bodies collected from two unpolluted and two polluted sites to (i) accumulate 53 elements and (ii) form selected LMWOAs, and finally, (iii) an evaluation of the possible relationship between elements and LMWOA content in mushrooms was made.

## Materials and methods

### Experimental material

Samples of *B. badius* fruiting bodies and underlying substrates (soils, flotation tailings) were collected between 20 September and 4 October 2015 from four sites located in central and southeastern Poland. Two of the sites were unpolluted areas of acidic sandy soils located in the Wielkopolska region (site 1 and site 2), while the other two sites were located in the Voivodeship of Lower Silesia (site 3 and site 4), where alkaline flotation tailings from copper production have been stored (Table 1). The used substrate (code 01 03 81) was the wastes from the flotational enrichment of non-ferrous metal ore other than presented in 01 03 80 in accordance with the Regulation of the Ministry of Economy (2015).

**Table 1:** Characteristics of sites where *Boletus badius* samples and underlying substrates were collected

Sample collection areas	GPS position	Elevation [m above sea level]
Site 1 unpolluted (forest area, soil)	52° 44′ 50.84″ N 17° 11′ 58.27″ E	85
Site 2 unpolluted (forest area, soil)	52° 42′ 02.17″ N 17° 06′ 00.70″ E	94
Site 3 polluted (flotation tailing)	51° 12′ 34.40″ N 15° 43′ 29.56″ E	235
Site 4 polluted (flotation tailing)	51° 12′ 38.96″ N 15° 40′ 51.30″ E	242

Fruiting bodies were collected from four, two, three, and two places within unpolluted site 1, unpolluted site 2, polluted site 3, and polluted site 4, respectively. Five collected fruiting bodies were analyzed from each of polluted and unpolluted site. As *B. badius* is an ectomycorrhizal species living in association with trees, the selection of sites was related to the presence of the same tree species. All sampled *B. badius* grew in association with *Pinus sylvestris* L. Samples of each underlying soil/flotation tailing (0.5 kg) were collected from the same locations as the collected mushrooms. Both soils/flotation tailings and mushroom fruiting bodies were transported immediately after collection to the laboratory for samples preparation and analysis.

### Determination of elements

#### Reagents

All used reagents were of analytical purity. Deionized ultrapure water was produced in a Milli-Q device (Millipore, Saint Luis, USA). Concentrated nitric acid (Merck, Darmstadt, Germany) was used for the preparation of samples and reference materials. ICP commercial analytical standards (Romil, England) were used for inductively coupled plasma optical emission spectrometry (ICP-OES) analysis. The standard reference materials CRM S-1—loess soil, CRM NCSDC (73349)—bush branches and leaves, and CRM 2709—soil were used for analysis quality control.

#### Procedure

Mushroom fruiting bodies were dried at 50 ± 2 °C for 96 h in an electric oven (SLW 53 STD, Pol-Eko, Wodzisław Śląski, Poland) and powdered in a laboratory Cutting Boll Mill PM 200 (Retsch GmbH, Haan, Germany). Accurately weighed 0.300 ± 0.001 g of each dry sample was digested with concentrated nitric acid in closed Teflon containers in the microwave digestion system Mars 5 Xpress (*CEM*, Matthews, NC, USA). Samples were then filtered through a paper filter (Qualitative Filter Papers, Whatman, grade 595 4–7 μm, UK) and diluted with water to a total volume of 15.0 mL. Each of the samples was analyzed in triplicate from the start of weighing. For each series of analysis, the reagent blank was analyzed. Soil/flotation tailing extraction by nitric acid was provided based on the scheme described above.

The pH determination was carried out for water extracts of solid samples with a direct electrochemical pH measurement obtained from a commercial pH meter. The redox potential determination was based on the measurement of the difference of potential between the platinum electrode and a standard hydrogen electrode (reference electrode). The electrochemical measurements of pH and redox potential including instrument calibrations were undertaken in accordance with ISO norms (ISO 10390 2005; ISO 11271 2002).

#### Instruments

The inductively coupled plasma optical emission spectrometer Agilent 5100 ICP-OES (Agilent, USA) was used for the determination of five major elements (Ca, K, Mg, Na, and P) and 47 trace elements (Ag, Al, As, Au, B, Ba, Bi, Cd, Ce, Co, Cr, Cu, Dy, Er, Eu, Fe, Ga, Gd, Ge, Ho, In, Ir, La, Li, Lu, Mn, Mo, Nd, Ni, Pb, Pd, Pr, Pt, Te, Rh, Ru, Sb, Sc, Se, Sm, Sr, Te, Tl, Tm, Y, Yb, and Zn). A synchronous vertical dual view (SVDV) of the plasma was accomplished using dichroic spectral combiner (DSC) technology, which allows axial and radial view analysis simultaneously. Common conditions were applied: radio frequency (RF) power 1.2 kW, nebulizer gas flow 0.7 L min^−1^, auxiliary gas flow 1.0 L min^−1^, plasma gas flow 12.0 L min^−1^, charge coupled device (CCD) temperature −40 °C, viewing height for radial plasma observation 8 mm, accusation time 5 s, three replicates.

Mercury concentration was measured with electrothermal atomic absorption spectrometry (ETAAS) and Zeeman background correction. A SpectrAA 280Z (Agilent Technologies, Australia) instrument equipped with pyrolytic graphite tubes and an Hg hollow cathode lamp (wavelength 253.7 nm, slit 0.5 nm, current 4 mA) was used. The temperature program was optimized: 55 s of drying at 85–120 °C, 8 s of ashing at 400 °C, and atomization at 1800 °C. As a chemical modifier, palladium solution (1000 mg L^−1^) was used in a volume of 10 μL for 20 μL of the sample. The limit of detection was found at the level of 0.01 mg kg^−1^, and uncertainty (measured as RSD) at a level of 5.0 % was obtained. Traceability was determined by the standard reference material analysis (mushroom material CS-M-I (LGC Standards, Poland) certified value 0.174 ± 0.018 mg kg^−1^, obtained value 0.16 ± 0.03 mg kg^−1^, recovery 94 %).

#### Analytical method validation

The detection limits were determined as 3-sigma criteria and were at the level of 0.01 mg kg^−1^ dry weight (DW) for all elements determined. The uncertainty for complete analytical process (including sample preparation) was at the level of 20 %. Traceability was checked using the reference materials listed above. The recovery (80–120 %) was acceptable for all elements determined, as presented in Supplementary data (Table S1). No certified values of concentration were available for several elements (Ag, Au, Bi, Ga, Ge, Ho, In, Ir, Pd, Pr, Pt, Re, Rh, Ru, Tl, Sm, Tm, Y, and Yb), and determined contents in the samples are presented in Supplementary data (Table S2–S4).

### Analysis of low-molecular-weight organic acids

#### Reagents

The LMWOA standards (acetic, citric, formic, fumaric, lactic, maleic, malic, malonic, oxalic, and succinic acids) were purchased from Supelco with a certified standard grade. Potassium dihydrogen phosphate (KH_2_PO_4_) and concentrated phosphoric acid (H_3_PO_4_) of analytical grade (Sigma) were used for pH adjustment of the mobile phase. Aqueous solution of the mobile phase was prepared from Milli-Q water (Millipore, Saint Luis, USA). Methanol was of HPLC grade, purchased from Sigma.

#### Procedure

Dried (at 50 ± 2 °C for 96 h) and powdered mushroom samples (5.0 g) were suspended in 20 mL deionized water (Milli-Q water, Millipore, Saint Luis, USA) and subjected to ultrasound action (400 W, 30 min, ambient temperature) using a 1500 W high-intensity ultrasonic processor (Sonorex Super RK 100H, Bandelin Electronic, Germany). The suspension was centrifuged at 3200*×g* for 15 min (Universal 320 R, Hettich, Tuttlingen, Germany), and the supernatant was filtered through a 0.45-μm cellulose membrane (Millipore) prior to analysis by reversed-phase column liquid chromatography (RPLC).

#### Instruments

RPLC was used for the separation and quantification of ten organic acids (acetic, citric, formic, fumaric, lactic, maleic, malic, malonic, oxalic, and succinic) in *B. badius* samples. RPLC analyses were conducted with a Waters Alliance 2695 Chromatograph coupled with a Waters 2996 Photodiode Array Detector (Waters Corp., Milford, MA, USA). Separation was performed on a Waters Atlantis C18 column (250 × 4.6 mm) with 5 μm particle size. RPLC conditions were as follows: mobile phase, 25 mM KH_2_PO_4_ adjusted to pH 2.5 with concentrated phosphoric acid and methanol (95:5, *v*/*v*); flow rate 0.8 mL min^−1^; UV detection wavelength 220 nm; at an ambient temperature of 25 ± 2 °C; and injection volume 10 μL. For the analysis of mushroom samples, gradient elution was used in every fifth sample to obtain 95 % methanol in 15 min to fully flush the column of hydrophobic compounds from previous injections (Cawthray 2003).

#### Analytical method validation

Analyzed organic acids were identified based on the retention time (*R*_t_) of original standards and their characteristic UV spectra and on the basis of external standard methods. For every analyzed organic acid, the calibration line equation and calibration coefficient, the limits of detection (LODs), and limits of quantification (LOQs) were calculated. LODs were calculated in a concentration range of the standards: 100–2000 μg mL^−1^ for oxalic, malic, malonic, lactic, citric, and succinic; 50–500 μg mL^−1^ for acetic; 10–100 μg mL^−1^ for formic; 0.05–0.50 μg mL^−1^ for fumaric; and 0.01–0.10 μg mL^−1^ for maleic, with an injection volume of 10 μL (Table S5).

### Statistical analysis

Statistical analysis was done using STATISTICA 10 and consisted of ANOVA followed by the post hoc Tukey’s test. The identical letters in rows, jointly for elements analyzed in samples (mushrooms, flotation tailings/soils) collected from both unpolluted and polluted areas, represent no differences at the significance level *α* = 0.05. Additionally, to determine correlation between element content in soil to their content in fruiting bodies (*r*_1_) and also between the element and total LMWOA contents in fruiting bodies (*r*_2_), the Pearson correlation coefficient (*r*) values were calculated.

## Results and discussion

### Concentration of elements in soils/flotation tailings

Data on elements in tested soils and flotation tailings are presented in Table 2 and in Supplementary data (Table S2) for elements with data on the informative value of their concentration. The concentration of most elements (excluding elements presented in Supplementary data) in both soils (sites 1 and 2) was similar, with the exception of Al, which was significantly lower than its level in flotation tailings.

**Table 2:** Content of elements [mg kg^−1^ DW] in tested soils/flotation tailings with pH and Eh values

Element	Unpolluted area		Polluted area	
	Site 1	Site 2	Site 3	Site 4
Ca	3896^c^ ± 148	4228^c^ ± 210	24652^a^ ± 3389	10976^b^ ± 847
K	6722^a^ ± 345	7268^a^ ± 253	3621^b^ ± 265	2054^c^ ± 158
Mg	271^c^ ± 22	309^c^ ± 13	4762^a^ ± 303	2326^b^ ± 120
Na	328^a^ ± 61	385^a^ ± 15	305^a^ ± 8	128^b^ ± 15
P	4236^a^ ± 119	4588^a^ ± 161	3625^b^ ± 105	3398^b^ ± 187
Al	497^d^ ± 32	892^c^ ± 37	4821^a^ ± 167	2965^b^ ± 229
As	0.21^c^ ± 0.04	0.43^c^ ± 0.07	21.4^a^ ± 2.9	8.80^b^ ± 0.70
B	0.98^c^ ± 0.18	0.65^c^ ± 0.10	54.6^a^ ± 7.5	10.4^b^ ± 0.8
Ba	16.0^c^ ± 3.0	14.0^c^ ± 2.0	141^a^ ± 19	37.0^b^ ± 3.0
Cd	0.24^c^ ± 0.04	0.31^c^ ± 0.05	0.51^b^ ± 0.07	1.33^a^ ± 0.10
Ce	3.76^c^ ± 0.70	2.08^c^ ± 0.33	18.9^a^ ± 2.60	8.87^b^ ± 0.68
Co	0.65^c^ ± 0.12	0.48^c^ ± 0.08	34.6^a^ ± 4.8	7.70^b^ ± 0.59
Cr	1.76^c^ ± 0.33	1.61^c^ ± 0.26	14.9^a^ ± 2.0	7.90^b^ ± 0.61
Cu	1.55^c^ ± 0.29	1.31^c^ ± 0.21	912^b^ ± 125	2143^a^ ± 142
Dy	0.11^b^ ± 0.02	0.14^b^ ± 0.02	1.31^a^ ± 0.18	0.09^b^ ± 0.01
Er	1.64^c^ ± 0.31	2.55^c^ ± 0.41	25.3^a^ ± 3.5	10.9^b^ ± 0.8
Eu	0.05^c^ ± 0.01	0.03^c^ ± 0.01	0.37^a^ ± 0.05	0.15^b^ ± 0.01
Fe	1313^c^ ± 86	1569^c^ ± 39	6599^a^ ± 529	3820^b^ ± 199
Gd	0.06^c^ ± 0.01	0.04^c^ ± 0.01	3.89^a^ ± 0.53	1.21^b^ ± 0.09
Hg	0.11^b^ ± 0.02	0.19^b^ ± 0.03	0.34^a^ ± 0.05	0.43^a^ ± 0.03
La	0.41^c^ ± 0.08	0.18^c^ ± 0.03	6.90^a^ ± 0.95	4.50^b^ ± 0.40
Li	0.42^c^ ± 0.08	0.61^c^ ± 0.10	21.0^a^ ± 2.9	10.7^b^ ± 0.8
Lu	0.04^b^ ± 0.01	0.03^b^ ± 0.01	0.29^a^ ± 0.04	0.08^b^ ± 0.01
Mn	23.0^c^ ± 4.0	25.0^c^ ± 4.0	1502^a^ ± 76	659^b^ ± 51
Mo	0.32^b^ ± 0.06	0.28^b^ ± 0.04	12.9^a^ ± 1.8	17.1^a^ ± 1.3
Nd	5.88^b^ ± 1.10	6.79^b^ ± 1.08	95.0^a^ ± 13.0	79.0^a^ ± 6.0
Ni	0.88^c^ ± 0.16	1.01^c^ ± 0.16	25.4^a^ ± 3.5	8.4^b^ ± 0.7
Pb	5.23^c^ ± 0.98	3.86^c^ ± 0.61	476^a^ ± 15	79.0^b^ ± 6.1
Sb	0.11^c^ ± 0.02	0.14^c^ ± 0.02	1.14^a^ ± 0.16	0.38^b^ ± 0.03
Sc	0.15^c^ ± 0.03	0.09^c^ ± 0.01	2.64^a^ ± 0.36	1.41^b^ ± 0.11
Se	0.03^c^ ± 0.01	0.02^c^ ± 0.01	0.21^b^ ± 0.03	0.65^a^ ± 0.05
Sr	3.02^c^ ± 056	5.00^c^ ± 0.79	517^a^ ± 18	206^b^ ± 16
Te	0.26^b^ ± 0.05	0.17^b^ ± 0.03	1.67^a^ ± 0.23	0.37^b^ ± 0.03
Zn	21.0^c^ ± 2.0	16.0^c^ ± 1.0	47.0^a^ ± 2.0	40.0^b^ ± 3.0
pH	5.03^c^ ± 0.10	4.78^c^ ± 0.17	7.99^b^ ± 0.08	8.17^a^ ± 0.12
Eh	551^a^ ± 19	516^b^ ± 25	386^c^ ± 43	361^c^ ± 32

Mean values (*n* = 5) ± standard deviations; identical superscripts denote significant (*p* < 0.05) difference between mean values in lines according to Tukey’s HSD test (ANOVA) for soils

No significant differences in the concentration of Mo, Nd, and P in flotation tailings collected from sites 3 and 4 were observed. The concentration of Cd, Cu, and Se in flotation tailings collected from site 4 was higher than from site 3, while the inverse situation was stated for the rest of the elements. It can be assumed that the concentration of the latter elements in *B. badius* fruiting bodies growing on flotation tailings at site 3 should be significantly higher than in bodies from site 4. It is worth noting that K and P concentration was significantly higher in soils than in flotation tailings. Moreover, no significant differences in Lu, Na, and Te concentration were observed between soils and tailings but irregularly among sites within both the underlying substrates.

### Content of elements in *B. badius* fruiting bodies

Data on the content of 53 elements in fruiting bodies of *B. badius* are presented in Table 3 and in Supplementary data (Table S3) for elements with information on the (informative) value of their concentration.

**Table 3:** Content of elements [mg kg^−1^ DW] in *Boletus badius* fruit bodies collected from unpolluted and polluted areas

Element	Unpolluted area		Polluted area	
	Site 1	Site 2	Site 3	Site 4
Ca	24^c^ ± 4	33^c^ ± 9	2706^a^ ± 166	2322^b^ ± 126
K	18932^a^ ± 640	17584^a^ ± 1178	1731^a^ ± 456	1968^a^ ± 1151
Mg	111^c^ ± 18	82^c^ ± 14	611^a^ ± 23	503^b^ ± 26
Na	273^a^ ± 28	226^a^ ± 21	148^b^ ± 5	121^b^ ± 20
P	4377^a^ ± 220	4401^a^ ± 299	3893^b^ ± 231	3602^b^ ± 318
Al	18^b^ ± 3	14^b^ ± 4	37^a^ ± 3	39^a^ ± 2
As	0.22^b^ ± 0.04	0.16^b^ ± 0.04	0.49^a^ ± 0.09	0.41^a^ ± 0.05
B	0.28^b^ ± 0.04	0.19^b^ ± 0.05	2.59^a^ ± 0.46	3.16^a^ ± 0.44
Ba	9.4^a^ ± 1.5	7.9^a^ ± 2.0	0.60^b^ ± 0.10	0.80^b^ ± 0.10
Cd	0.24^a^ ± 0.04	0.19^a^ ± 0.05	0.30^a^ ± 0.06	0.26^a^ ± 0.04
Ce	0.11^b^ ± 0.02	0.11^b^ ± 0.03	1.95^a^ ± 0.37	1.64^a^ ± 0.23
Co	0.03^b^ ± 0.02	0.04^b^ ± 0.01	0.19^a^ ± 0.04	0.15^a^ ± 0.02
Cr	0.14^a^ ± 0.02	0.18^a^ ± 0.05	0.18^a^ ± 0.03	0.13^a^ ± 0.02
Cu	14^a^ ± 2	17^a^ ± 4	13^a^ ± 2	9^a^ ± 1
Dy	0.03^b^ ± 0.01	0.04^b^ ± 0.01	0.07^a^ ± 0.01	0.09^a^ ± 0.01
Er	0.03^b^ ± 0.01	0.04^b^ ± 0.01	0.49^a^ ± 0.09	0.55^a^ ± 0.08
Eu	0.01^b^ ± 0.01	0.01^b^ ± 0.01	0.02^a^ ± 0.01	0.02^a^ ± 0.01
Fe	24^a^ ± 4	29^a^ ± 7	28^a^ ± 5	35^a^ ± 2
Gd	0.05^b^ ± 0.01	0.02^c^ ± 0.01	0.05^b^ ± 0.01	0.09^a^ ± 0.01
Hg	0.32^b^ ± 0.05	0.21^b^ ± 0.05	0.89^a^ ± 0.17	0.96^a^ ± 0.13
La	0.09^ab^ ± 0.01	0.11^a^ ± 0.03	0.08^ab^ ± 0.02	0.06^b^ ± 0.01
Li	0.06^a^ ± 0.01	0.08^a^ ± 0.01	0.03^b^ ± 0.01	0.04^b^ ± 0.01
Lu	0.01^b^ ± 0.01	0.01^b^ ± 0.01	0.02^a^ ± 0.01	0.02^a^ ± 0.01
Mn	11.3^a^ ± 1.8	14.3^a^ ± 3.7	1.80^b^ ± 0.30	1.40^b^ ± 0.20
Mo	0.04^b^ ± 0.01	0.03^b^ ± 0.01	0.15^a^ ± 0.03	0.11^a^ ± 0.02
Nd	0.05^b^ ± 0.01	0.03^b^ ± 0.01	9.38^a^ ± 0.68	7.71^a^ ± 1.08
Ni	0.37^a^ ± 0.06	0.29^ab^ ± 0.07	0.26^ab^ ± 0.05	0.19^b^ ± 0.03
Pb	0.11^c^ ± 0.02	0.17^b^ ± 0.04	0.59^a^ ± 0.11	0.64^a^ ± 0.09
Sb	0.03^b^ ± 0.01	0.02^b^ ± 0.01	0.31^a^ ± 0.06	0.24^a^ ± 0.03
Sc	0.03^c^ ± 0.01	0.04^bc^ ± 0.01	0.07^a^ ± 0.01	0.06^ab^ ± 0.01
Se	0.02^b^ ± 0.01	0.01^b^ ± 0.01	2.78^a^ ± 0.53	3.54^a^ ± 0.50
Sr	0.19^b^ ± 0.03	0.15^b^ ± 0.04	2.65^a^ ± 0.51	1.94^a^ ± 0.27
Te	0.02^b^ ± 0.01	0.01^b^ ± 0.01	0.33^a^ ± 0.06	0.26^a^ ± 0.04
Zn	72^b^ ± 4	88^ab^ ± 13	109^ab^ ± 12	86^a^ ± 6

Mean values (*n* = 5) ± standard deviations; identical superscripts denote significant (*p* < 0.05) difference between mean values in lines according to Tukey’s HSD test (ANOVA) for whole fruit bodies

Based on the results of statistical testing, four groups of elements (with the exception of elements presented in Supplementary data) were indicated as regards their content in mushroom bodies from unpolluted and polluted areas. The largest group I consists of 19 elements with significantly higher content in fruiting bodies from polluted areas than from unpolluted ones. This group comprised Al, As, B, Ca, Ce, Co, Dy, Er, Eu, Hg, Lu, Mg, Mo, Nd, Pb, Sb, Se, Sr, and Te. An inverse situation was observed in five elements (Ba, Li, Mn, Na, and P) forming group II. Kojta et al. (2012) reported the same observation for Na and P which would suggest that these elements accumulate in a different way. For the five elements (Cd, Cr, Cu, Fe, and K) forming group III, no significant differences were stated in the contents of *B. badius* bodies collected from unpolluted and polluted areas. Group IV consists of the remaining five elements (Gd, La, Ni, Sc, and Zn), the contents of which vary irregularly among sites from unpolluted and polluted areas. Data of Table 3 suggest that there is a greater accumulation of most of the determined elements in fruiting bodies from polluted rather than from unpolluted areas, although five elements forming group II were accumulated at greater levels in fruiting bodies growing in unpolluted areas. Such results together with observations for the groups III and IV suggest that the content of elements in underlying substrate (soil/flotation tailing) is not the only factor affecting the rate of accumulation in fruiting bodies.

Due to differing levels of elements in the underlying substrates, there arises a question regarding the possible correlation between element contents in soil/flotation tailings and their content in fruiting bodies (*r*_1_). Such a positive significant correlation was observed for 19 elements (Al, As, Ca, Ce, Co, Er, Eu, Hg, Lu, Mg, Mo, Nd, Pb, Sb, Sc, Se, Sr, Te, and Zn), collated in Table 4. Negative significant correlation was determined for Ba (−0.6792), Cu (−0.7050), and Mn (−0.8046). It is known that element accumulation in fruiting bodies does not simply depend on element content in substrate. Numerous papers have pointed to the significant role of environmental factors related with soil composition and its traits, although these factors are not clearly elucidated (Borovička and Řanda 2007; Campos and Tejera 2011; Falandysz and Bielawski 2007; Falandysz and Borovička 2013). A decrease of pH level is generally associated with increasing bioavailability of numerous elements, enabling their higher accumulation in plants (Blake and Goulding 2002; Tyler and Olsson 2001). Unfortunately, such relationships have not yet been defined for mushrooms.

**Table 4:** Pearson correlation coefficients (*r*) for particular elements calculated between element content in underlying substrates to their content in *Boletus badius* fruit bodies (*r*_1_) and between element content and total low-molecular-weight organic acids content in fruit bodies (*r*_2_)

Element	*r*_1_	*r*_2_	Element	*r*_1_	*r*_2_
Ca	0.8602*	0.9726*	Fe	0.2059	0.3994
K	−0.3117	0.0711	Gd	0.2225	0.6798
Mg	0.9439*	0.9843*	Hg	0.8589*	0.9618*
Na	−0.5225	−0.8217*	La	−0.5404	−0.8179*
P	0.5389	−0.5917*	Li	−0.4578	−0.2685
Al	0.8848*	0.9754*	Lu	0.6216*	0.9664*
As	0.8835*	0.9820*	Mn	−0.8046*	−0.9780*
B	0.5680	0.9255*	Mo	0.8414*	0.9840*
Ba	−0.6792*	−0.9250*	Nd	0.9847*	0.9752*
Cd	0.2727	0.9487*	Ni	−0.3522	−0.6152*
Ce	0.8571*	0.9740*	Pb	0.6260*	0.9180*
Co	0.8047*	0.9654*	Sb	0.8314*	0.9825*
Cr	0.1475	−0.0996	Sc	0.8290*	0.9134*
Cu	−0.7050*	−0.7903*	Se	0.8719*	0.9154*
Dy	0.2429	0.8234*	Sr	0.9308*	0.9778*
Er	0.7623*	0.9351*	Te	0.7428*	0.9816*
Eu	0.8218*	0.7017	Zn	0.6495*	0.6534

*Significant at *p* < 0.05

### Profile of LMWOAs in mushroom

**Fig. 1:**
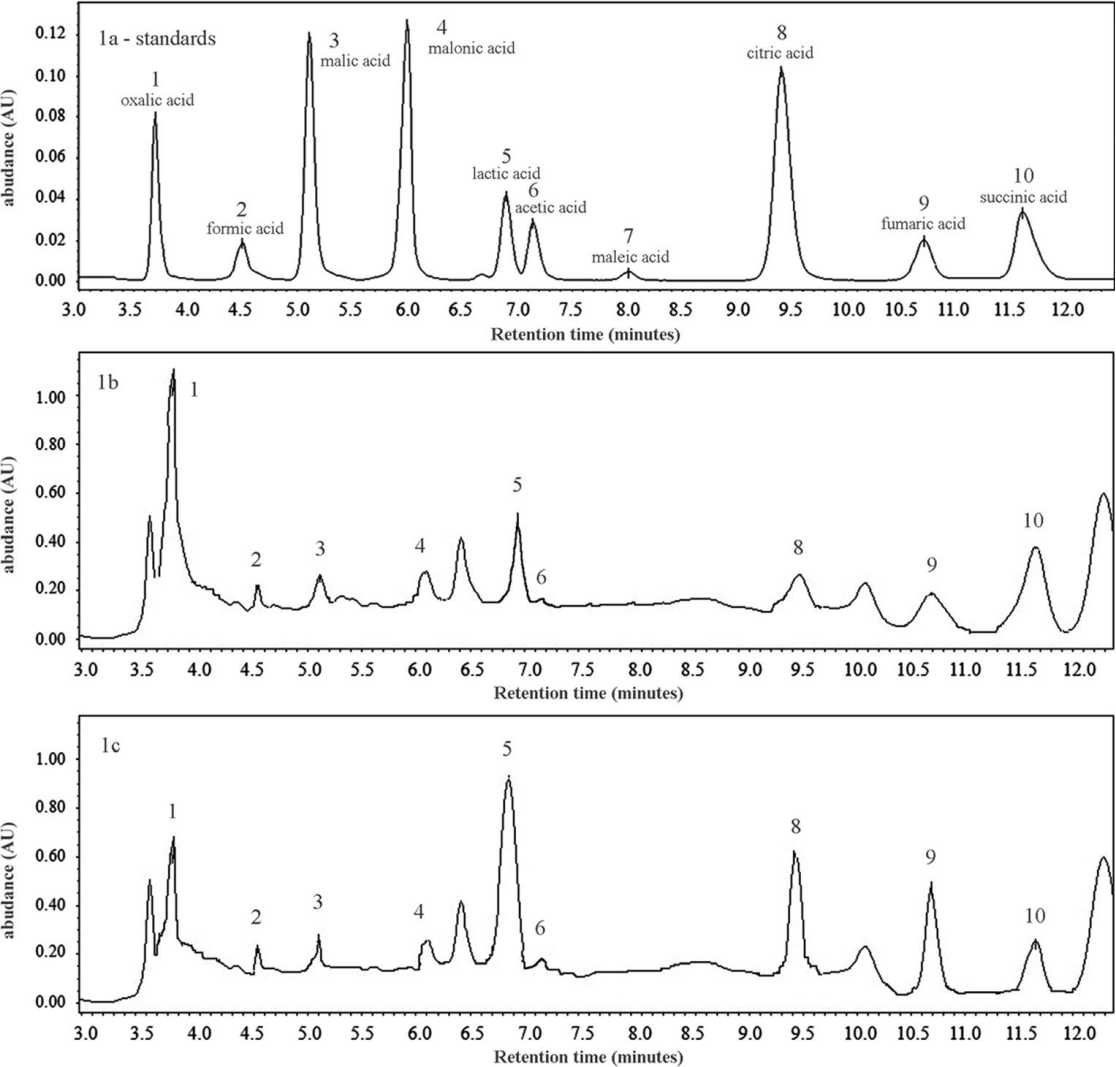
Chromatograms of low-molecular-weight organic acids of standards (**a**) and in mushrooms collected from unpolluted (**b**) and polluted (**c**) areas. Peaks: oxalic acid (*1*), formic acid (*2*), malic acid (*3*), malonic acid (*4*), lactic acid (*5*), acetic acid (*6*), maleic acid (*7*), citric acid (*8*), fumaric acid (*9*), and succinic acid (*10*)

Nine of the ten studied organic acids were determined by RPLC analysis. Maleic acid content was below LOD. Chromatograms of LMWOAs in mushrooms collected from unpolluted and polluted areas are shown in Fig. 1. The standards of analyzed LMWOAs are presented in Fig. 1a. The main peaks in Fig. 1b (unpolluted area) belonged to lactic acid and succinic acid, while in Fig. 1c (polluted area), next to them, a markedly elevated content of citric acid can be seen. No significant differences between the areas were observed in the contents of the remaining six organic acids (Table 5).

**Table 5:** Low-molecular-weight organic acid content and composition (mg g^−1^ DW) in *Boletus badius* fruit bodies collected from unpolluted and polluted areas

Organic acids	Unpolluted area		Polluted area	
	Site 1	Site 2	Site 3	Site 4
Acetic acid	0.04^b^ ± 0.00	0.03^b^ ± 0.01	0.07^a^ ± 0.01	0.07^a^ ± 0.01
Citric acid	0.50^b^ ± 0.050	0.56^b^ ± 0.203	5.41^a^ ± 0.596	4.61^a^ ± 0.29
Formic acid	0.18^ab^ ± 0.02	0.15^ab^ ± 0.01	0.19^a^ ± 0.03	0.12^b^ ± 0.02
Fumaric acid	0.01^a^ ± 0.00	0.17^a^ ± 0.11	0.02^a^ ± 0.00	0.02^a^ ± 0.00
Lactic acid	7.17^b^ ± 0.61	9.60^b^ ± 1.53	11.2^ab^ ± 1.7	14.8^a^ ± 2.0
Maleic acid	nd	nd	nd	nd
Malic acid	0.43^b^ ± 0.04	0.43^b^ ± 0.08	0.06^a^ ± 0.01	0.06^a^ ± 0.01
Malonic acid	0.95^a^ ± 0.09	1.05^a^ ± 0.24	0.26^b^ ± 0.05	0.29^b^ ± 0.04
Oxalic acid	1.37^a^ ± 0.14	1.01^b^ ± 0.01	0.74^b^ ± 0.07	0.37^c^ ± 0.11
Succinic acid	8.88^a^ ± 0.333	5.30^b^ ± 0.38	4.23^b^ ± 0.60	2.88^c^ ± 0.67
Total content of identified acids	19.5^a^ ± 1.1	18.3^a^ ± 2.5	22.2^a^ ± 1.4	23.2^a^ ± 3.2

Mean values (*n* = 5) ± standard deviations; identical superscripts denote significant (*p* < 0.05) difference between mean values in lines according to Tukey’s HSD test (ANOVA) for whole fruit bodies

*nd* not detected

To the best of our knowledge, information on organic acid content and composition in *B. badius* fruiting bodies has been lacking to date. Fragmentary data for related species of the genus *Boletus* are available (Barros et al. 2013; Fernandes et al. 2014; Leal et al. 2013; Ribeiro et al. 2008). Nevertheless, they deal particularly with citric, malic, and oxalic acids, whereas for several of the other acids collated in Table 5, there is little information. Generally, the reported contents vary widely and are higher (except for succinic acid) than the levels of Table 5.

The average content (*n* = 5) of lactic acid in fruiting bodies from the unpolluted sites 1 and 2 was 7.17 ± 0.61 and 9.60 ± 1.53 mg g^−1^ DW, respectively (Table 5), while respective contents of succinic acid were 8.88 ± 0.33 and 5.30 ± 0.38 mg g^−1^ DW. In the polluted sites 3 and 4, contents of lactic acid increased to 11.2 ± 1.7 and 14.8 ± 2.0 mg g^−1^ DW, while those of succinic acid decreased to 4.23 ± 0.61 and 2.89 ± 0.67 mg g^−1^ DW as compared the sites 1 and 2. Further, the content of citric acid in fruiting bodies from the polluted sites increased about tenfold when compared to the level from the unpolluted sites. Such a considerable increase may have an essential influence on the inherent ability to accumulate an excess of essential metals and non-essential metal ions as well (Böke et al. 2015). Biosynthesis of citric acid, an intermediate in the tricarboxylic acid cycle, can occur by way of glyoxylate oxidation (Gadd 1999). Citric acid may thus play an important role in metal homeostasis (Böke et al. 2015; Gadd 1999; López-Bucio et al. 2000) and with its metal complex-forming abilities provide essential metals and anionic nutrients for *B. badius*. Therefore, mushrooms may have an ecological advantage and be able to grow fairly well under different environmental conditions, e.g., metal contamination (Zeppa et al. 2012) or at very low pH values (Karaffa et al. 2001). However, as in the case of plants (Ghnaya et al. 2013; Magdziak et al. 2013), the composition and content of organic acids in fruiting bodies may depend on both mushroom species and environmental conditions.

### Relationship of element content and LMWOA profile

An analysis of the relationship between element contents and total content of LMWOAs in mushrooms as a Pearson correlation coefficient (*r*_2_) are collated in Table 4 and in Supplementary data (Table S4) for elements with (informative) information on the value of their concentration.

A positive significant correlation was observed for 20 elements, namely Al, As, B, Ca, Cd, Ce, Co, Dy, Er, Hg, Lu, Mg, Mo, Nd, Pb, Sb, Sc, Se, Sr, and Te, while a negative significant correlation was found for 7 elements (Ba, Cu, La, Mn, Na, Ni, and P). A high proportion of positive correlation seems to confirm the important role of LMWOAs in element accumulation known in plants (Drzewiecka et al. 2014; Kutrowska and Szelag 2014; Goliński et al. 2015). Pearson correlation coefficients (*r*) between the individual organic acid and element contents were also estimated (Table 6). According to the obtained results, a highly positive correlation (*r* = 0.900) with some elements (Al, As, B, Ca, Ce, Co, Er, Hg, Lu, Mg, Mo, Na, Nd, Pb, Sb, Sc, Se, Sr, and Te) is especially exhibited by citric acid and oxalic acid to Al, B, Ce, Er, Nd, Pb, and Se, while malonic and malic acids show a highly positive correlation with Ba and Mn. On the other hand, a highly negative correlation was observed between malic acid and Al, B, Ca, Ce, Er, Hg, Mg, Nd, Pb, Sb, Se, Sr, and Te; malonic acid and Ca, Mg, and Nd; and citric acid and Ba and Mn. However, this initial survey of relations between numerous elements and LMWOAs content in fruiting bodies of one mushroom species will need further comprehensive research.

**Table 6:** Pearson correlation coefficients (*r*) between content of particular low-molecular-weight organic acid and element in *Boletus badius* fruit bodies

Element	Low-molecular-weight organic acid									
	Acetic	Citric	Formic	Fumaric	Lactic	Malic	Malonic	Oxalic	Succinic	Total
Al	0.9069*	0.9635*	−0.1123	−0.4725	0.6901*	−0.9250*	−0.8964*	−0.7508*	−0.6786*	0.6761*
As	0.8311*	0.9200*	0.2294	−0.4394	0.4274	−0.8150*	−0.7910*	−0.5133	−0.4755	0.5736
B	0.9012*	0.9480*	−0.2458	−0.4121	0.7603*	−0.9362*	−0.8912*	−0.8418*	−0.7536*	0.6701*
Ba	−0.8236*	−0.9368*	0.1988	0.2956	−0.7043*	0.9506*	0.9027*	0.8726*	0.8107*	−0.5543
Ca	0.8963*	0.9940*	−0.0653	−0.395	0.6749*	−0.9607*	−0.9246*	−0.7897*	−0.7371*	0.6299*
Cd	0.6742*	0.6782*	0.3184	−0.4529	0.2390	−0.5317	−0.5239	−0.2109	−0.1745	0.5092
Ce	0.9007*	0.9905*	−0.0234	−0.3852	0.6391*	−0.9373*	−0.8950*	−0.7567*	−0.7048*	0.6242*
Co	0.8863*	0.9754*	0.0543	−0.3448	0.6028*	−0.8945*	−0.8505*	−0.6993*	−0.6792*	0.6114*
Cr	0.0179	0.0606	0.3911	0.2976	−0.1477	0.1174	0.1433	0.1753	−0.0290	−0.0717
Cu	−0.4822	−0.5202	0.3766	0.4049	−0.5130	0.6525*	0.6443*	0.6323*	0.4468	−0.3852
Dy	0.8729*	0.8694*	−0.3241	−0.2923	0.7967*	−0.8375*	−0.7782*	−0.8344*	−0.7774*	0.6547*
Er	0.9163*	0.9691*	−0.1896	−0.3834	0.7423*	−0.9419*	−0.8933*	−0.8369*	−0.7623*	0.6617*
Eu	0.5073	0.6455*	0.5470	−0.1891	0.0530	−0.4832	−0.4708	−0.1451	−0.2220	0.2756
Fe	0.5178	0.4429	−0.3837	−0.0009	0.5763*	−0.3772	−0.3163	−0.5416	−0.5733	0.3806
Gd	0.7379*	0.6418*	−0.3567	−0.5461	0.6465*	−0.6578*	−0.6370*	−0.5966*	−0.4146	0.6502*
Hg	0.8844*	0.9406*	−0.1737	−0.4652	0.7008*	−0.9162*	−0.8809*	−0.7667*	−0.6793*	0.6693*
K	0.2679	0.0412	−0.3146	−0.0805	0.3771	0.0057	0.0439	−0.1392	0.0601	0.4669
La	−0.5123	−0.5516	0.3357	0.4467	−0.5039	0.6748*	0.6693*	0.6211*	0.4293	−0.4094
Li	−0.2105	−0.3864	0.1812	−0.3759	−0.3709	0.4113	0.3496	0.5828*	0.7184*	−0.0041
Lu	0.8990*	0.9303*	−0.0548	−0.3910	0.6454*	−0.8522*	−0.8083*	−0.6943*	−0.6435*	0.6562*
Mg	0.8864*	0.9890*	−0.0163	−0.4147	0.6492*	−0.9474*	−0.9170*	−0.7444*	−0.6953*	0.6384*
Mn	−0.8253*	−0.9095*	0.1385	0.4898	−0.6267*	0.9339*	0.9082*	0.7647*	0.6330*	−0.5964*
Mo	0.8669*	0.9665*	0.1303	−0.4075	0.5207	−0.8836*	−0.8507*	−0.6309*	−0.5919*	0.5922*
Na	0.8257*	0.9377*	0.0634	−0.4254	0.5515	−0.8744*	−0.8518*	−0.6249*	−0.5637	0.6245*
Nd	0.9003*	0.9934*	−0.0314	−0.3925	0.6697*	−0.9518*	−0.9149*	−0.7647*	−0.7156*	0.6461*
Ni	−0.4919	−0.5733	0.5347	0.0324	−0.6944*	0.6880*	0.6347*	0.8622*	0.8066*	−0.3218
P	−0.4590	−0.5565	0.5478	−0.0187	−0.6906*	0.6599*	0.6049*	0.8630*	0.8310*	−0.2926
Pb	0.9101*	0.9627*	−0.1878	−0.3311	0.7470*	−0.9232*	−0.8693*	−0.8373*	−0.7855*	0.6499*
Sb	0.8894*	0.9865*	0.0386	−0.3996	0.5926*	−0.9242*	−0.8862*	−0.7117*	−0.6624*	0.6119*
Sc	0.8274*	0.9037*	0.0507	−0.2260	0.5697	−0.7956*	−0.7437*	−0.6492*	−0.6737*	0.5547
Se	0.9118*	0.9497*	−0.2463	−0.3979	0.7666*	−0.9362*	−0.8880*	−0.8521*	−0.7619*	0.6712*
Sr	0.8793*	0.9844*	0.0590	−0.3835	0.5775*	−0.9189*	−0.8807*	−0.7027*	−0.6616*	0.5976*
Te	0.8930*	0.9887*	0.0194	−0.3995	0.6063*	−0.9309*	−0.8920*	−0.7264*	−0.6742*	0.6159*
Zn	0.5709	0.6731*	0.2972	0.1973	0.3570	−0.4731	−0.3995	−0.3913	−0.4932	0.4010

*Significant at *p* < 0.05

## Conclusions

The accumulation of elements in mushroom species is diverse as regards the place (element concentration in substrate), where fruiting bodies are grown. The obtained results have revealed the existence of relationships between content of elements and low-molecular-weight organic acids in *B. badius*. The significantly higher content of the majority of elements in mushrooms growing on flotation tailings than in soil was related with higher acid contents thereby proving that these acids play a significant role in element uptake. The higher content of citric acids in fruiting bodies growing on the polluted substrate allows us to state that mushrooms are able to grow in a polluted area; therefore, a high level of citric acid content in substrate could be associated with a more effective removal of elements from the environment, e.g., using plants.

## Electronic supplementary material

Table S1 Traceability studies for certified reference materials: recoveries in % (DOCX 16 kb)

Table S2 Content of elements [mg kg-1 DW] with informative value of their concentration in tested soils/flotation tailings (DOCX 17 kb)

Table S3 Content of elements [mg kg-1 DW] with informative value of their concentration in *Boletus badius* fruit bodies collected from unpolluted and polluted areas (DOCX 17 kb)

Table S4 Pearson correlation coefficients (r) for particular elements with informative value of their concentration, calculated between element content in underlying substrates to their content in *Boletus badius* fruit bodies (r1), and between element content and total low-molecular-weight organic acids content in fruit bodies (r2) (* - significant at *p* < 0.05) (DOCX 15 kb)

Table S5 Analytical characteristic of the reversed-phase column liquid chromatography method used for organic acids analysis (DOCX 15 kb)
