# Supplementary material for: Content of selected elements and low-molecular-weight organic acids in fruiting bodies of edible mushroom Boletus badius (Fr.) Fr. from unpolluted and polluted areas
Source: Environ Sci Pollut Res Int. 2016 Jul 28;23(20):20609–18. doi: 10.1007/s11356-016-7222-z (PMC5099368; doi:10.1007/s11356-016-7222-z)
Supplement: Supplementary file 1 — Traceability studies for certified reference materials: recoveries in % (DOCX 16 kb) [file 11356_2016_7222_MOESM1_ESM.docx]

**Supplementary data**

**Table S1.** Traceability studies for certified reference materials: recoveries in %

| Element | A | B | C | Element | A | B | C |
| --- | --- | --- | --- | --- | --- | --- | --- |
| Al | x | 37* | 27* | Li | x | 115 | x |
| As | 91 | 77 | 115 | La | 87 | 78 | 60* |
| B | x | 90 | 64* | Lu | 87 | x | 117 |
| Ba | 83 | 74 | x | Mg | 88 | 87 | 60* |
| Ca | 79 | 91 | 63* | Mn | 74 | 95 | 80 |
| Cd | 113 | 104 | 116 | Mo | x | 94 | x |
| Ce | 83 | 88 | 94 | Na | 92 | 88 | 46* |
| Co | 86 | 89 | 78 | Nd | x | 91 | 113 |
| Cr | 97 | 86 | 84 | Ni | 92 | 95 | 96 |
| Cu | 86 | 79 | x | Pb | 90 | 94 | 94 |
| Dy | x | x | 115 | Sb | 90 | 116 | 101 |
| Er | x | x | x | Sc | 90 | x | 94 |
| Eu | 87 | x | 82 | Se | x | x | 97 |
| Fe | 79 | 77 | 88 | Sr | 109 | 106 | x |
| Gd | x | 118 | 112 | Te | x | x | 112 |
| K | 80 | 94 | 45* | Zn | 105 | 95 | 81 |

x – not certified; A -CRM S-1; B - CRM NCSDC; C - CRM San Jaoqain 2709;

*low recovery value
