# Supplementary material for: Content of selected elements and low-molecular-weight organic acids in fruiting bodies of edible mushroom Boletus badius (Fr.) Fr. from unpolluted and polluted areas
Source: Environ Sci Pollut Res Int. 2016 Jul 28;23(20):20609–18. doi: 10.1007/s11356-016-7222-z (PMC5099368; doi:10.1007/s11356-016-7222-z)
Supplement: Supplementary file 2 — Content of elements [mg kg-1 DW] with informative value of their concentration in tested soils/flotation tailings (DOCX 17 kb) [file 11356_2016_7222_MOESM2_ESM.docx]

**Table S2.** Content of elements [mg kg^-1^ DW] with informative value of their concentration in tested soils/flotation tailings

| Element | Unpolluted area | | Polluted area | | |
| --- | --- | --- | --- | --- | --- |
|  | Site 1 | Site 2 | | Site 3 | Site 4 |
| Ag | 0.22^c^±0.04 | 0.31^c^±0.05 | | 13.2^a^±1.8 | 4.62^b^±0.35 |
| Au | 0.16^c^±0.03 | 0.11^c^±0.02 | | 1.42^a^±0.20 | 0.51^b^±0.04 |
| Bi | 0.14^c^±0.03 | 0.11^c^±0.02 | | 0.81^a^±0.11 | 0.31^b^±0.02 |
| Ga | 0.02^c^±0.01 | 0.02^c^±0.01 | | 2.40^a^±0.33 | 0.80^b^±0.10 |
| Ge | 0.02^c^±0.01 | 0.03^bc^±0.01 | | 0.05^b^±0.01 | 0.16^a^±0.01 |
| Ho | 0.03^b^±0.01 | 0.02^b^±0.01 | | 0.12^a^±0.02 | 0.03^b^±0.01 |
| In | 0.02^c^±0.01 | 0.01^c^±0.01 | | 0.38^a^±0.05 | 0.16^b^±0.01 |
| Ir | 0.12^c^±0.02 | 0.08^c^±0.01 | | 3.94^a^±0.54 | 1.66^b^±0.13 |
| Pd | 0.01^b^±0.01 | 0.01^b^±0.01 | | 0.08^a^±0.01 | 0.08^a^±0.01 |
| Pr | 0.16^b^±0.03 | 0.12^b^±0.02 | | 0.68^a^±0.09 | 0.81^a^±0.06 |
| Pt | 0.07^c^±0.01 | 0.05^c^±0.01 | | 60.7^a^±8.3 | 32.1^b^±2.5 |
| Re | 0.02^c^±0.01 | 0.02^c^±0.01 | | 0.17^b^±0.02 | 0.26^a^±0.02 |
| Rh | 0.01^c^±0.01 | 0.02^c^±0.01 | | 0.26^a^±0.04 | 0.17^b^±0.01 |
| Ru | 0.26^a^±0.05 | 0.15^b^±0.02 | | 0.11^b^±0.02 | 0.33^a^±0.03 |
| Sm | 0.02^b^±0.01 | 0.03^b^±0.01 | | 0.13^a^±0.02 | 0.12^a^±0.01 |
| Tl | 0.04^c^±0.01 | 0.02^c^±0.01 | | 1.44^a^±0.20 | 0.69^b^±0.05 |
| Tm | 0.15^c^±0.03 | 0.20^c^±0.03 | | 0.88^a^±0.12 | 0.48^b^±0.04 |
| Y | 0.09^c^±0.02 | 0.07^c^±0.01 | | 7.60^a^±1.04 | 4.10^b^±0.30 |
| Yb | 0.10^c^±0.02 | 0.14^c^±0.02 | | 0.64^a^±0.09 | 0.31^b^±0.02 |
