# Supplementary material for: Content of selected elements and low-molecular-weight organic acids in fruiting bodies of edible mushroom Boletus badius (Fr.) Fr. from unpolluted and polluted areas
Source: Environ Sci Pollut Res Int. 2016 Jul 28;23(20):20609–18. doi: 10.1007/s11356-016-7222-z (PMC5099368; doi:10.1007/s11356-016-7222-z)
Supplement: Supplementary file 3 — Content of elements [mg kg-1 DW] with informative value of their concentration in Boletus badius fruit bodies collected from unpolluted and polluted areas (DOCX 17 kb) [file 11356_2016_7222_MOESM3_ESM.docx]

**Table S3.** Content of elements [mg kg^-1^ DW] with informative value of their concentration in *Boletus badius* fruit bodies collected from unpolluted and polluted areas

| Element | Unpolluted area | | Polluted area | |
| --- | --- | --- | --- | --- |
|  | Site 1 | Site 2 | Site 3 | Site 4 |
| Ag | 0.18^b^±0.06 | 0.26^ab^±0.07 | 0.31^ab^±0.06 | 0.38^a^±0.05 |
| Au | 0.01^c^±0.01 | 0.01^c^±0.01 | 0.05^b^±0.01 | 0.08^a^±0.01 |
| Bi | 0.02^c^±0.01 | 0.02^c^±0.01 | 0.68^a^±0.13 | 0.51^b^±0.07 |
| Ga | 0.01^b^±0.01 | 0.01^b^±0.01 | 0.04^a^±0.01 | 0.03^a^±0.01 |
| Ge | 0.01^c^±0.01 | 0.01^c^±0.01 | 0.36^a^±0.06 | 0.24^b^±0.03 |
| Ho | 0.01^c^±0.01 | 0.03^ab^±0.01 | 0.04^a^±0.01 | 0.02^bc^±0.01 |
| In | 0.02^b^±0.01 | 0.01^b^±0.01 | 1.31^a^±0.24 | 1.06^a^±0.15 |
| Ir | 0.03^b^±0.01 | 0.02^b^±0.01 | 0.90^a^±0.17 | 0.68^a^±0.10 |
| Pd | 0.01^c^±0.01 | 0.02^c^±0.01 | 0.08^a^±0.02 | 0.05^b^±0.01 |
| Pr | 0.02^b^±0.01 | 0.04^ab^±0.01 | 0.05^a^±0.01 | 0.03^b^±0.01 |
| Pt | 0.01^b^±0.01 | 0.01^b^±0.01 | 0.79^a^±0.13 | 0.88^a^±0.12 |
| Re | 0.02^bc^±0.01 | 0.01^c^±0.01 | 0.04^a^±0.01 | 0.04^a^±0.01 |
| Rh | 0.03^b^±0.01 | 0.04^b^±0.01 | 0.08^a^±0.02 | 0.07^a^±0.01 |
| Ru | 0.02^c^±0.01 | 0.01^c^±0.01 | 0.04^b^±0.01 | 0.06^a^±0.01 |
| Sm | 0.01^c^±0.01 | 0.01^c^±0.01 | 0.04^b^±0.01 | 0.07^a^±0.01 |
| Tl | 0.01^b^±0.01 | 0.02^a^±0.01 | 0.02^a^±0.01 | 0.01^b^±0.01 |
| Tm | 0.02^b^±0.01 | 0.02^b^±0.01 | 0.04^a^±0.01 | 0.02^b^±0.01 |
| Y | 0.01^c^±0.01 | 0.01^c^±0.01 | 0.05^a^±0.01 | 0.03^b^±0.01 |
| Yb | 0.01^a^±0.01 | 0.01^a^±0.01 | 0.01^a^±0.01 | 0.01^a^±0.01 |

Mean values (n=5) ± standard deviations; identical superscripts denote significant (p < 0.05) difference between mean values in lines according to Tukey's HSD test (ANOVA) for whole fruit bodies.
