# Supplementary material for: Content of selected elements and low-molecular-weight organic acids in fruiting bodies of edible mushroom Boletus badius (Fr.) Fr. from unpolluted and polluted areas
Source: Environ Sci Pollut Res Int. 2016 Jul 28;23(20):20609–18. doi: 10.1007/s11356-016-7222-z (PMC5099368; doi:10.1007/s11356-016-7222-z)
Supplement: Supplementary file 4 — Pearson correlation coefficients (r) for particular elements with informative value of their concentration, calculated between element content in underlying substrates to their content in Boletus badius fruit bodies (r1), and between element content and total low-molecular-weight organic acids content in fruit bodies (r2) (* - significant at p < 0.05) (DOCX 15 kb) [file 11356_2016_7222_MOESM4_ESM.docx]

**Table S4.** Pearson correlation coefficients (r) for particular elements with informative value of their concentration, calculated between element content in underlying substrates to their content in *Boletus badius* fruit bodies (r_1_), and between element content and total low-molecular-weight organic acids content in fruit bodies (r_2_) (* - significant at p < 0.05)

| Element | r_1_ | r_2_ |
| --- | --- | --- |
| Ag | 0.4050 | 0.6761 |
| Au | 0.4938 | 0.8301 |
| Bi | 0.8464* | 0.9762* |
| Ga | 0.7350* | 0.6241 |
| Ge | 0.5823* | 0.9761* |
| Ho | 0.6523* | 0.3984 |
| In | 0.8801* | 0.9768* |
| Ir | 0.9059* | 0.9782* |
| Pd | 0.8667* | 0.9193* |
| Pr | 0.2606 | 0.4035 |
| Pt | 0.8750* | 0.9416* |
| Re | 0.6899* | 0.9784* |
| Rh | 0.8352* | 0.8352* |
| Ru | 0.4848 | 0.8458* |
| Tl | 0.2433 | -0.0429 |
| Sm | 0.8407* | 0.7906* |
| Tm | 0.8292* | 0.7175 |
| Y | 0.9445* | 0.9369* |
| Yb | -0.0752 | -0.3762 |
