# Supplementary material for: Content of selected elements and low-molecular-weight organic acids in fruiting bodies of edible mushroom Boletus badius (Fr.) Fr. from unpolluted and polluted areas
Source: Environ Sci Pollut Res Int. 2016 Jul 28;23(20):20609–18. doi: 10.1007/s11356-016-7222-z (PMC5099368; doi:10.1007/s11356-016-7222-z)
Supplement: Supplementary file 5 — Analytical characteristic of the reversed-phase column liquid chromatography method used for organic acids analysis (DOCX 15 kb) [file 11356_2016_7222_MOESM5_ESM.docx]

**Table S5.** Analytical characteristic of the reversed-phase column liquid chromatography method used for organic acids analysis

| Organic acids | R_t_ (retention time) | Calibration equationa (Regression) | Coefficient (R^2^) | Limit | |
| --- | --- | --- | --- | --- | --- |
|  | minutes |  |  | LOD (μg/mL) | LOQ (μg/mL) |
| Acetic | 7.1 | y = 230x - 280 | 0.9987 | 12.6 | 25.2 |
| Citric | 9.4 | y = 584x - 104 | 0.9999 | 9.5 | 19.0 |
| Formic | 4.5 | y = 616x - 732 | 0.9991 | 9.2 | 18.4 |
| Fumaric | 10.7 | y = 221x + 365 | 0.9710 | 0.09 | 0.18 |
| Lactic | 6.9 | y = 170x + 286 | 0.9900 | 9.4 | 18.8 |
| Maleic | 8.0 | y = 807x - 457 | 0.9995 | 0.1 | 0.2 |
| Malic | 5.1 | y = 383x + 794 | 0.9999 | 4.8 | 9.6 |
| Malonic | 6.0 | y = 440x + 128 | 0.9999 | 9.4 | 18.8 |
| Oxalic | 3.7 | y = 199x - 205 | 0.9885 | 9.9 | 19.8 |
| Succinic | 11.6 | y = 237x - 118 | 0.9967 | 9.6 | 19.2 |
